# Dual effects of leptin in perioperative gas exchange of morbidly obese patients

**DOI:** 10.1371/journal.pone.0199610

**Published:** 2018-07-05

**Authors:** Michele Carron, Giovanna Ieppariello, Gabriele Martelli, Giulia Gabellini, Mirto Foletto, Egle Perissinotto, Carlo Ori

**Affiliations:** 1 Department of Medicine—DIMED, Section of Anesthesiology and Intensive Care, University of Padova, Padova, Italy; 2 Department of Surgical, Oncological and Gastroenterological Sciences, Section of Surgery, University of Padova, Padova, Italy; 3 Department of Cardiac, Thoracic and Vascular, Unit of Biostatistics, Epidemiology and Public Health, University of Padova, Padova, Italy; Auburn University College of Veterinary Medicine, UNITED STATES

## Abstract

Leptin has shown positive effects on respiratory function in experimental settings. The role of leptin on perioperative respiratory function in morbidly obese patients has not been established. We performed a retrospective analysis of morbidly obese patients undergoing laparoscopic sleeve gastrectomy. Fasting serum leptin and interleukin (IL)-6 were measured preoperatively, and arterial blood gases were obtained pre- and postoperatively. Outcome variables were arterial partial pressure of oxygen (PaO_2_), arterial partial pressure of carbon dioxide (PaCO_2_), and differences in PaO_2_ and PaCO_2_ between pre- and postoperative values (ΔPaO_2_, ΔPaCO_2_; postoperative minus preoperative). Patients with lower (<40 μg/L) and higher (≥40 μg/L) leptin levels were compared. Bravais-Pearson’s correlation, multiple linear regression, and logistic regression analysis were performed. A total of 112 morbidly obese patients were included. Serum leptin was significantly higher in females than in males (42.86±12.89 vs. 30.67±13.39 μg/L, p<0.0001). Leptin was positively correlated with body mass index (*r* = 0.238; p = 0.011), IL-6 (*r* = 0.473; p<0.0001), and ΔPaO_2_ (*r* = 0.312; p = 0.0008). Leptin was negatively correlated with preoperative PaO_2_ (*r* = -0.199; p = 0.035). Preoperative PaO_2_ was lower, ΔPaCO_2_ was smaller, and ΔPaO_2_ was greater in the high leptin group than in the low leptin group. In multiple regression analysis, leptin was negatively associated with preoperative PaO_2_ (estimate coefficient = -0.147; p = 0.023). In logistic regression analysis, leptin was associated with improved ΔPaO_2_ (odds ratio [OR] = 1.104; p = 0.0138) and ΔPaCO_2_ (OR = 0.968; p = 0.0334). Leptin appears to have dual effects related to perioperative gas exchange in obese patients undergoing bariatric surgery. It is associated with worse preoperative oxygenation but improved respiratory function after surgery.

## Introduction

The incidence and prevalence of obesity continue to increase globally [1]. Obesity has major importance because of its strong association with morbidity and all-cause mortality [2–4]. Obesity reflects an imbalance between food intake and energy expenditure, leading to excessive accumulation of adipose tissue [5].

Adipose tissue is not only the main storage site for surplus food energy, but it is also an endocrine organ [5]. It produces bioactive substances, called adipokines or adipocytokines, that initiate chronic low-grade inflammation and affect numerous processes in various organs. Among these substances, leptin seems to have an integral role in morbid obesity [6]. Leptin is a 16-kD protein encoded by the *ob* gene that interacts with receptors in the hypothalamus to inhibit eating. Its importance is clearly illustrated by the extreme obesity observed in the *ob/ob* mouse (C57BL/6J-*Lep*^ob^), which cannot produce functional leptin [7].

Leptin may also have respiratory effects. Evidence from animal models suggests that leptin stimulates ventilation. Acute leptin replacement significantly increases baseline ventilation [7–9]. Leptin microinjections into the nucleus tractus solitarius of rat brains is associated with increased ventilation, suggesting that leptin may act directly through the respiratory control center [7–9]. Studies in several animal models, such as rats, mice, and baboons, identified leptin receptors in the lungs, suggesting that these organs are also a target for leptin-mediated signaling [7–9].

Obesity has well-established effects on respiratory function, which are exacerbated by supine positioning, surgery, and anesthesia. Arterial blood gases are often abnormal in obesity, characterized by hypoxemia and, less frequently, hypercapnia [10–12]. Data are lacking regarding the potential respiratory effects of leptin in obese patients. Examining pulmonary effects is complicated, as it can be difficult to distinguish the role of leptin from the effects of obesity, as well as the biology of adipose tissue [7]. High leptin levels have been associated with hypoxemia in obstructive sleep apnea-hypopnea syndrome (OSAHS) and obesity hypoventilation syndrome (OHS) [13,14]. Leptin has not been heretofore investigated in the perioperative period. The goal of this study was to explore the potential role of leptin on perioperative respiratory function, assessed by gas exchange analysis, in patients with morbid obesity undergoing bariatric surgery.

## Materials and methods

### Population

We conducted a retrospective evaluation using our hospital database and medical records of morbidly obese patients who underwent laparoscopic sleeve gastrectomy under general anesthesia at our institution. We included only patients with obstructive sleep apnea (OSA) in whom fasting serum leptin was measured preoperatively and arterial blood gases were measured preoperatively and postoperatively. Patients were recruited consecutively until the sample size was achieved. An equal allocation strategy was used to include adequate numbers of patients with high (≥40 μg/L) and low (<40 μg/L) serum leptin values. We chose 40 μg/L as the cut-off based on previous studies [14,15]. No other exclusion criteria were applied.

### Anesthesia

All patients underwent a standardized general anesthetic. Anesthesia was induced with propofol 2 mg/kg lean body weight (LBW), ketamine 1 mg/kg LBW, and fentanyl 3–4 μg/kg LBW, and neuromuscular blockade was achieved with rocuronium 1 mg/kg LBW [16]. After tracheal intubation, the patients' lungs were ventilated with a 35/65 oxygen/air mixture using a pressure-regulated volume-control mode (FLOW-i Ventilator, MAQUET Medical System, Italy). The expiratory tidal volume was maintained at 8 mL/kg LBW, and the respiratory rate was adjusted to keep the partial arterial carbon dioxide pressure (PaCO_2_) at 35–40 mm Hg. Lung recruitment maneuvers were performed after tracheal intubation and immediately before tracheal extubation. Anesthesia was maintained with desflurane to ensure a bispectral index value of approximately 40. At the conclusion of surgery, sugammadex 2 mg/kg total body weight was administered to ensure full reversal (train-of-four ratio ≥1.0) of moderate neuromuscular blockade. Ketoprofen 100 mg and ondansetron 8 mg were also administered at this time to reduce postoperative pain, as well as nausea and vomiting.

### Endpoints

Preoperative and postoperative arterial partial pressure of oxygen (PaO_2_) and PaCO_2_ were the primary outcome variables. Arterial blood was obtained for gas exchange analysis 15 minutes before anesthesia induction and 15 minutes after tracheal extubation. We computed changes in PaO_2_ and PaCO_2_ from before to after surgery as follows: ΔPaO_2_ = PaO_2_^POST^–PaO_2_^PRE^ and ΔPaCO_2_ = PaCO_2_^POST^–PaCO_2_^PRE^. Sex, age, and body mass index (BMI) before surgery were recorded as potential confounding variables.

Fasting serum leptin concentration was measured within 1 month before surgery. As previous research indicated that leptin participates in inflammation modulation, promotes the production of pro-inflammatory cytokines [5], and is a predictor of interleukin (IL)-6 in obese juveniles [17], serum IL-6 concentrations were measured in each serum sample to explore the relationship between leptin and IL-6.

### Blood samples

Arterial blood was obtained for gas exchange analysis using a small caliber needle to minimize patient discomfort. Gas exchange analysis was performed immediately after sampling using the Rapidlab^®^1200 System (Siemens Healthcare Diagnostics Ltd., Camberley, UK).

Venous blood sample for leptin and IL-6 was collected into a 10-mL vacutainer before noon, after patients underwent a 12-hour overnight fast. The sample was maintained in a polystyrene container with ice packs and brought quickly to the laboratory for analysis; all analyses were performed within 2 hours of blood collection. Leptin was measured by radioimmunoassay (Mediagnost^®^, Reutlingen, Germany) according to the routine methodology used in our institution’s laboratory. The analytical sensitivity of the assay was 0.1 ng/mL and the intra-assay coefficient of variability was <5%. IL-6 was measured by electrochemiluminescence immunoassay (Immulite 1, Siemens, UK England) according to our laboratory’s standard procedures. The intra-assay coefficient of variability was <7%.

### Statistical analysis

The sample size was based on prior studies. A difference in preoperative PaO_2_ of 5 mm Hg between low and high leptin groups was previously observed and considered clinically relevant for predicting postoperative hypoxemia in obese patients [18,19]. PaO_2_ was assumed to be normally distributed, with a standard deviation homoscedastic between groups and equal to 10 mm Hg; the type I error was set as 0.05 and the type II error as 0.2; and data were anticipated to be missing for approximately 10% of patients. Considering these assumptions and settings, the sample size was calculated as 112 patients, divided equally between low and high leptin groups.

Descriptive analysis was used to summarize patient characteristics. Normality of distribution of quantitative characteristics was analyzed using the Shapiro-Wilk test. Pre- and postoperative variables were compared using the paired t-test if the variable was normally distributed or the Wilcoxon signed rank test if it was non-normally distributed. To determine the strength and direction of association between two variables, we used Bravais-Pearson’s correlation test for normally distributed variables and Spearman's rank correlation test for variables that were not normally distributed.

Continuous variables are presented as mean ± standard deviation (SD) and 95% confidence interval (CI). The two-tail Student’ t-test or two-tail Mann-Whitney U test was used to compare low to high leptin groups for variables normally or non-normally distributed variables, respectively. Median, minimum, and maximum values are reported for non-normally distributed variables. Sex distribution is presented as number (percentage) and compared between groups using the chi-square test.

We used multiple linear regression analysis to determine the relationship between one dependent normally distributed variable and one or more independent normally distributed variables. For non-normally distributed variables, we first dichotomized continuous variables, then conducted logistic regression analysis to determine odds ratios (ORs) with 95% CIs.

All statistical analyses were conducted using R version 3.4.0 (2017-04-21). *P-*values <0.05 were considered statistically significant.

### Ethical statement

All procedures in the study were performed in accordance with the ethical standards of our institutional research committee and the 1964 Helsinki declaration and its later amendments. Formal consent was not necessary for this type of study (the data were analyzed retrospectively and anonymously). The Ethics Committee for Clinical Research of Padova approved this study.

## Results

The characteristics of the 112 consecutive morbidly obese patients included in this study are summarized in Table 1. There were more females than males (69 vs. 43, p<0.001). BMI did not differ between females and males (44.54±6.23 vs. 45.16±5.06 kg/m^2^, p = 0.583). Females had significantly higher serum leptin levels (42.86±12.89 vs. 30.67±13.39 μg/L, p<0.0001) and PaO_2_^POST^ (82.91±11.96 vs. 77.02±8.60 mm Hg, p = 0.006) than males. No other significant sex-related differences were observed.

**Table 1:** Characteristics of 112 morbidly obese patients included in the study.

	Min	Q1	Median	Q3	Max	IQR	Mean	SD	95% CI
**Age (years)**	24	42.8	47	52	68	9.3	46.9	8.3	45.3, 48.4
**BMI (kg/m**^**2**^**)**	34.7	41	44.8	47.7	66.2	6.7	44.8	5.8	43.7, 45.9
**Serum leptin (μg/L)**	12	28	40	48	88	20	38.2	14.3	35.5, 40.9
**Serum IL-6 (ng/L)**	1.5	2.5	3.4	4.3	11.3	1.82	3.8	1.9	3.4, 4.1
**PaO**_**2**_^**PRE**^ **(mm Hg)**	53.6	71. 8	78	85.8	100.3	14.0	78.7	9.1	77.0, 80.4
**PaO**_**2**_^**POST**^ **(mm Hg)**	51.4	73.0	79.5	87.1	112.2	14.2	80.7^**a**^	11.1	78.6, 82.7
**ΔPaO**_**2**_ **(mm Hg)**	-63.9	-5.9	0.05	7.8	65	13.8	0.13	17.3	-3.1, 3.37
**PaCO**_**2**_^**PRE**^ **(mm Hg)**	24.7	34.8	38.1	40.8	51.8	6.0	37.8	4.2	37.0, 38.5
**PaCO**_**2**_^**POST**^ **(mm Hg)**	23.2	37.0	40.1	42.9	50.1	6.0	39.7^**b**^	5.2	38.8, 40.7
**ΔPaCO**_**2**_ **(mm Hg)**	-28.2	-5.4	0.85	8.5	39.9	13.9	1.94	11.7	-0.24, 4.12

BMI, body mass index; CI, confidence interval; IL-6, interleukin 6; IQR, interquartile range; max, maximum; min, minimum; PaCO_2_^POST^, postoperative arterial partial pressure of carbon dioxide (PaCO_2_); PaCO_2_^PRE^, preoperative PaCO_2;_ ΔPaCO_2_, PaCO_2_^POST^ minus PaCO_2_^PRE^; PaO_2_^POST^, postoperative arterial partial pressure of oxygen (PaO_2_); PaO_2_^PRE^, preoperative PaO_2_; ΔPaO_2_, PaO_2_^POST^ minus PaO_2_^PRE^; Q1, first quartile; Q3, third quartile; SD, standard deviation.

^a^ p = 0.080 for PaO_2_^POST^ vs. PaO_2_^PRE^ (paired t-test)

^b^ p<0.0001 for PaCO_2_^POST^ vs. PaCO_2_^PRE^ (paired t-test)

**Fig 1:**
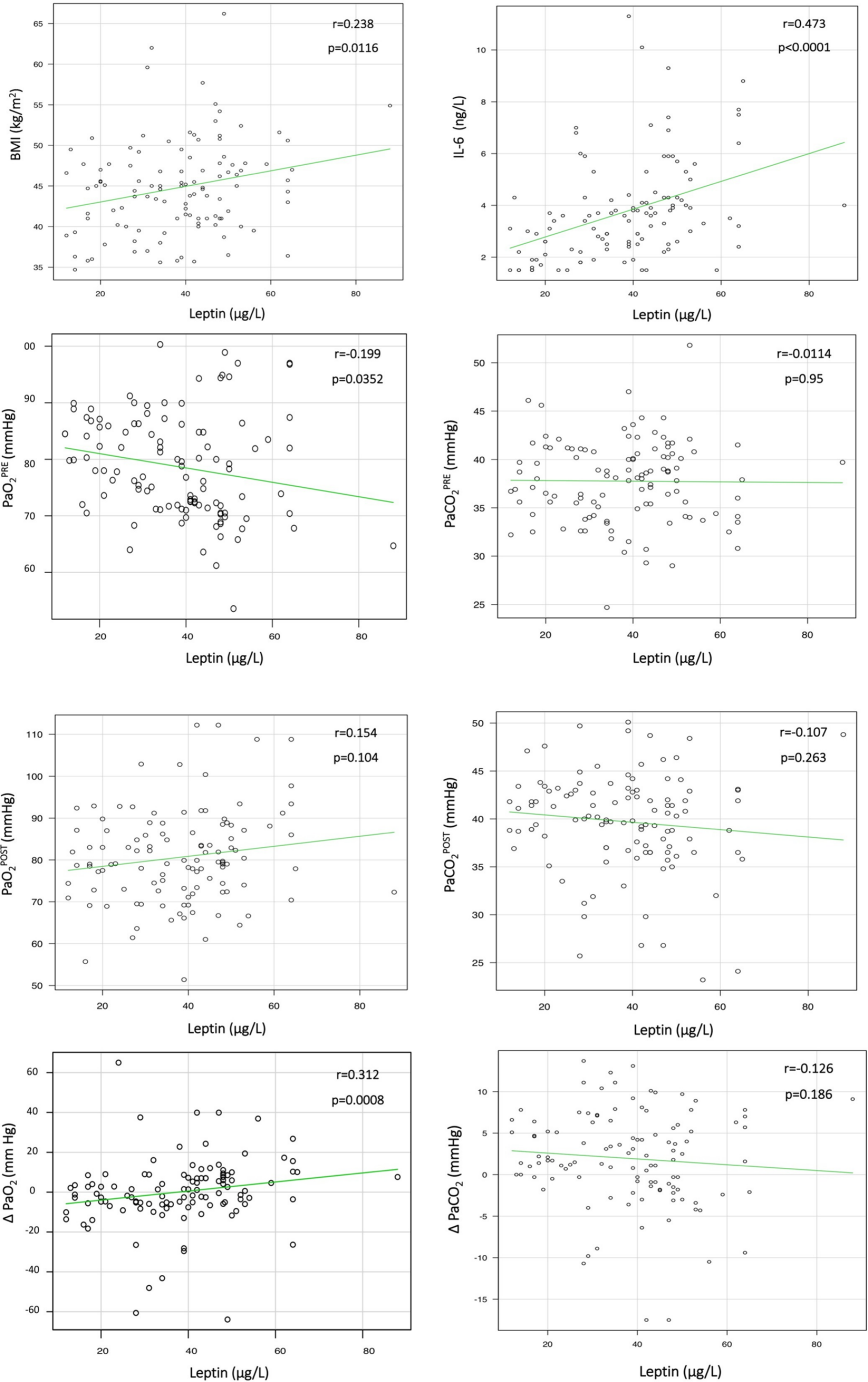
Correlation between serum leptin and other variables. As shown, leptin was positively correlated with BMI, serum IL-6, PaO_2_^POST^, and ΔPaO_2_ and negatively correlated with PaO_2_^PRE^, PaCO_2_^PRE^, PaCO_2_^POST^, and ΔPaCO_2_. BMI, body mass index; IL-6, interleukin 6; PaCO_2_^POST^, postoperative arterial partial pressure of carbon dioxide (PaCO_2_); PaCO_2_^PRE^, preoperative PaCO_2_; ΔPaCO_2_, PaCO_2_^POST^ minus PaCO_2_^PRE^; PaO_2_^POST^, postoperative arterial partial pressure of oxygen (PaO_2_); PaO_2_^PRE^, preoperative PaO_2_; ΔPaO_2_, PaO_2_^POST^ minus PaO_2_^PRE^; r, Bravais-Pearson correlation coefficient.

**Fig 2:**
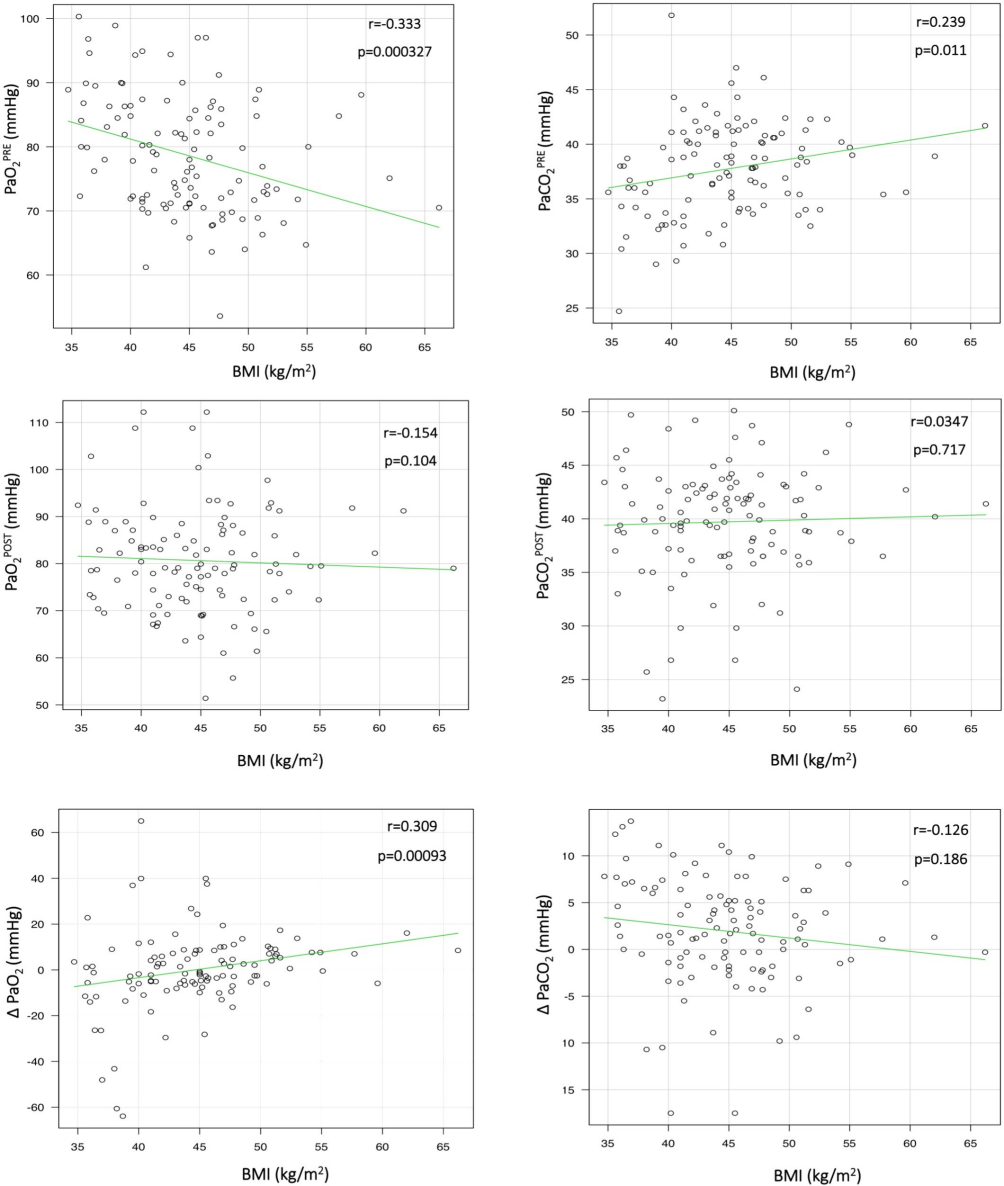
Correlation between body mass index and other variables. As shown, BMI was positively correlated with PaCO_2_^PRE^, PaCO_2_^POST^, and ΔPaO_2_ and negatively correlated with PaO_2_^PRE^, PaO_2_^POST^, and ΔPaCO_2_. BMI, body mass index; PaCO_2_^POST^, postoperative arterial partial pressure of carbon dioxide (PaCO_2_); PaCO_2_^PRE^, preoperative PaCO_2_; ΔPaCO_2_, PaCO_2_^POST^ minus PaCO_2_^PRE^; PaO_2_^POST^, postoperative arterial partial pressure of oxygen (PaO_2_); PaO_2_^PRE^, preoperative PaO_2_; ΔPaO_2_, PaO_2_^POST^ minus PaO_2_^PRE^; r, Bravais-Pearson correlation coefficient.

In the total population, serum leptin correlated significantly with BMI, serum IL-6, PaO_2_^PRE^, and ΔPaO_2_ (Fig 1). BMI was significantly correlated with PaO_2_^PRE^, PaCO_2_^PRE^, and ΔPaO_2_ (Fig 2).

Characteristics of the high leptin and low groups are shown in Table 2. The high leptin group had a significantly lower PaO_2_^PRE^, larger ΔPaO_2_, and smaller ΔPaCO_2_ than the low leptin group. No other significant differences in gas exchange parameters were observed between groups.

**Table 2:** Characteristics of high and low serum leptin groups.

Variable		Leptin (μg/L)		p-value
		<40 (n = 56)	≥40 (n = 56)	
**Sex (%)**	Male	27 (48.2)	16 (28.6)	0.051
	Female	29 (51.8)	40 (71.4)	
**Age (y)**	Mean (SD) [95% CI]	47.12 (8.77) [44.77, 49.46]	46.61 (7.8) [44.52, 48.69]	0.742
**BMI (kg/m**^**2**^**)**	Mean (SD) [95% CI]	43.62 (5.64) [42.11, 45.13]	45.94 (5.7) [44.41, 47.46]	0.033
**Serum leptin (μg/L)**	Mean (SD) [95% CI]	26.77 (8.41) [24.51, 29.02]	49.6 (8.83) [47.23, 51.96]	<0.001
**Serum IL-6 (ng/L)**	Mean (SD) [95% CI]	3.14 (1.72) [2.67, 3.60]	4.37 (1.95) [3.84, 4.89]	<0.001
**PaO**_**2**_^**PRE**^ **(mm Hg)**	Mean (SD) [95% CI]	80.97 (7.32) [79.01, 82.93]	76.44 (10.2) [70.20, 79.67]	0.008
**PaO**_**2**_^**POST**^ **(mm Hg)**	Mean (SD) [95% CI]	78.85 (10.5) [76.03, 81.66]	82.44 (11.5) [79.36, 85.52]	0.088
**ΔPaO**_**2**_ **(mm Hg)**	Mean (SD) [95% CI]	-2.12 (9.49) [-4.66, 0.42]	5.99 (12.17) [3.44, 8.53]	<0.001
	Median [min, max]	-3.45 [-28.20, 25.50]	5.95 [-26.40, 39.90]	
**PaCO**_**2**_^**PRE**^ **(mm Hg)**	Mean (SD) [95% CI]	37.35 (4.2) [36.22, 38.47]	38.15 (4.1) [37.05, 39.24]	0.317
**PaCO**_**2**_^**POST**^ **(mm Hg)**	Mean (SD) [95% CI]	40.47 (4.7) [39.21, 41.72]	38.97 (5.5) [38.49, 41.44]	0.128
**ΔPaCO**_**2**_ **(mm Hg)**	Mean (SD) [95% CI]	3.11 (5.19) [1.72, 4.5]	0.82 (6.03) [-0.79, 2.43]	0.0372
	Median [min, max]	2.85 [-10.70, 13.70]	0.8 [-17.50, 10.10]	

BMI, body mass index; CI, confidence interval; IL-6, interleukin 6; max, maximum; min, minimum; PaCO_2_^POST^, postoperative arterial partial pressure of carbon dioxide (PaCO_2_); PaCO_2_^PRE^, preoperative PaCO_2_; ΔPaCO_2_, PaCO_2_^POST^ minus PaCO_2_^PRE^; PaO_2_^POST^, postoperative arterial partial pressure of oxygen (PaO_2_); PaO_2_^PRE^, preoperative PaO_2_; ΔPaO_2_, PaO_2_^POST^ minus PaO_2_^PRE^; SD, standard deviation.

During multiple regression analysis, male sex (p = 0.021), BMI (p = 0.004), and serum leptin (p = 0.023) were negatively associated with PaO_2_^PRE^, and male sex (p = 0.0059) was negatively associated with PaO_2_^POST^. Age (p = 0.005) and BMI (p = 0.003) were positively associated with PaCO_2_^PRE^. No variables were significantly associated with PaCO_2_^POST^ (Table 3).

**Table 3:** Multiple linear regression analysis to explain the relationship between gas exchange values and variables considered.

Variables		Regression model							Fitted regression model					
Dependent	Independent	VIF	EC	SE	t- value	p-value	AR^2^	p-value	EC	SE	t- value	p-value	AR^2^	p-value
**PaO**_**2**_^**PRE**^	Sex (M)	1.257	-4.239	1.84	-2.291	0.024	0.138	<0.001	-4.287	1.83	-2.339	0.021	0.145	<0.001
	Age	1.044	0.025	0.09	-0.260	0.794			-	-	-	-		
	BMI	1.117	-0.426	0.14	-2.899	0.004			-0.420	0.14	-2.906	0.004		
	Leptin	1.318	-0.148	0.06	-2.292	0.024			-0.147	0.06	-2.295	0.023		
**PaO**_**2**_^**POST**^	Sex (M)	1.257	-5.115	2.379	-2.149	0.0338	0.037	0.089	-5.885	2.098	-2.804	0.0059	0.058	0.006
	Age	1.044	0.004	0.128	-0.036	0.971			-	-	-	-		
	BMI	1.117	-0.103	0.189	-0.547	0.585			-	-	-	-		
	Leptin	1.318	0.057	0.083	0.687	0.493			-	-	-	-		
**PaCO**_**2**_^**PRE**^	Sex (M)	1.257	1.539	0.856	1.798	0.074	0.128	<0.001	1.423	0.767	1.856	0.066	0.136	<0.001
	Age	1.044	0.131	0.046	2.845	0.006			0.130	0.045	2.848	0.005		
	BMI	1.117	0.191	0.068	2.816	0.005			0.197	0.065	3.033	0.003		
	Leptin	1.318	0.009	0.029	0.310	0.757			-	-	-	-		
**PaCO**_**2**_^**POST**^	Sex (M)	1.257	0.778	1.138	0.683	0.496	-0.017	0.712	-	-	-	-	-	-
	Age	1.044	-0.006	0.061	-0.105	0.916			-	-	-	-		
	BMI	1.117	0.045	0.090	0.498	0.619			-	-	-	-		
	Leptin	1.318	-0.032	0.040	-0.812	0.418			-	-	-	-		

Multiple linear regression analysis was performed to explain the relationship between one dependent normally distributed variable (gas exchange parameters) and independent normally distributed variables (sex, age, BMI, and serum leptin). Multicollinearity was not detected using variance inflation factors. Using the Akaike information criterion, backward/forward stepwise regression analysis was then performed to choose the best model. BMI, body mass index; EC, estimate coefficient; PaCO_2_^POST^, postoperative arterial partial pressure of carbon dioxide (PaCO_2_); PaCO_2_^PRE^, preoperative PaCO_2_; ΔPaCO_2_, PaCO_2_^POST^ minus PaCO_2_^PRE^; PaO_2_^POST^, postoperative arterial partial pressure of oxygen (PaO_2_); PaO_2_^PRE^, preoperative PaO_2_; ΔPaO_2_, PaO_2_^POST^ minus PaO_2_^Pre^; SE, standard error; VIF, variance inflation factor.

In logistic regression analysis, serum leptin (p = 0.013) and BMI (p = 0.024) were significantly associated with ΔPaO_2_, whereas only leptin (p = 0.021) was significantly associated with ΔPaCO_2_ (Table 4).

**Table 4:** Logistic regression analysis between leptin and BMI and variation of gas exchange parameters.

**Variables**		**Regression model**					**Fitted regression model**			
**Dependent**	**Independent**	**VIF**	**OR**	**L95%**	**U95%**	**p-value**	**OR**	**L95%**	**U95%**	**p-value**
ΔPaO_2_	Sex (M)	1.239	1.020	0.409	2.540	0.9670	-	-	-	-
	Age	1.040	1.010	0.964	1.060	0.6190	-	-	-	-
	BMI	1.069	1.090	1.010	1.180	0.0248	1.090	1.010	1.180	0.0246
	Leptin	1.238	1.040	1.010	1.070	0.0218	1.040	1.010	1.070	0.0138
ΔPaCO_2_	Sex (M)	1.235	0.752	0.296	1.910	0.5480	-	-	-	-
	Age	1.063	0.966	0.917	1.020	0.1840	-	-	-	-
	BMI	1.108	0.974	0.904	1.050	0.4820	-	-	-	-
	Leptin	1.280	0.962	0.930	0.995	0.0231	0.966	0.938	0.995	0.0210

Logistic regression analysis was performed to explain the relationship between one dependent non-normally distributed variable (ΔPaO_2_ and ΔPaCO_2_) and independent normally distributed variables (sex, age, BMI, and serum leptin). Multicollinearity was not detected using variance inflation factors. Using the Akaike information criterion, backward/forward stepwise regression analysis was performed to choose the best model. BMI, body mass index; L95%, lower limit of the 95% confidence interval (CI); OR, odds ratio; PaCO_2_^POST^, postoperative arterial partial pressure of carbon dioxide (PaCO_2_); PaCO_2_^PRE^, preoperative PaCO_2_; ΔPaCO_2_, PaCO_2_^POST^ minus PaCO_2_^PRE^; PaO_2_^POST^, postoperative arterial partial pressure of oxygen (PaO_2_); PaO_2_^PRE^, preoperative PaO_2_; ΔPaO_2_, PaO_2_^POST^ minus PaO_2_^PRE^; U95%, upper limit of the 95% CI; VIF, variance inflation factor.

## Discussion

This study demonstrated that leptin (as well as BMI and male sex) was negatively associated with preoperative respiratory function—mainly associated with a reduced PaO_2_—and positively associated with postoperative respiratory function—as evidenced by improved gas exchange.

Obesity is associated with worse gas exchange compared with normal-weight individuals [20]. Even in the current study involving only morbidly obese patients, higher BMI was associated with a lower PaO_2_^PRE^, higher PaCO_2_^PRE^, and greater ΔPaO_2_. Zavorsky et al. found lower PaO_2_ (mean 81, range 50–95 mm Hg), normal PaCO_2_, and higher alveolar-to-arterial oxygen partial pressure difference (P[A-a]O_2_ 23, range 5–38 mm Hg) in morbidly obese patients. Furthermore, morbidly obese men had poorer gas exchange at rest than morbidly obese women [21]. Among 42 morbidly obese subjects scheduled for bariatric surgery, a 10-mm Hg PaO_2_ difference and an 8-mm Hg P(A-a)O_2_ difference were observed between sexes, with women exhibiting superior gas exchange [22]. In obesity, adipose tissue accumulation in the chest wall and thoracic cavity, increased pulmonary blood volume, and excessive abdominal contents pushing the diaphragm upward decrease lung volume and compliance [23–26] and lead to atelectasis, ventilation-perfusion mismatch, increased intrapulmonary shunting [11,23], and heterogeneous airway narrowing [27]. These changes culminate in arterial hypoxemia and sometimes hypercapnia [26–29].

Adipose tissue can also affect respiratory function through pro-inflammatory adipokines, such as leptin [5,30]. Airway epithelial cells express leptin receptors, suggesting that visceral and locally released leptin may affect airway function (as observed in animal models) and contribute to airway remodeling in obese people [30]. Additionally, leptin may indirectly act on airways through pro-inflammatory cytokines produced by inflammatory cells, such as tumor necrosis factor [TNF]-α and IL-6 [7,24,30–33]. TNF-α promotes airway inflammation and reactivity, increases airway smooth muscle cell contractility, and may be implicated in airway remodeling [34]. In the current study, we observed a positive correlation between serum leptin levels and serum IL-6 levels. IL-6 enhances airway inflammation and affects the function of numerous non-inflammatory cells in the lung (e.g., epithelial cells, endothelial cells, smooth muscle cells, fibroblasts), acting directly and via other cytokines (e.g., IL-4 and IL-13) [35]. Interestingly, hypoxemia increases IL-6; this increase seems to be independent of pro-inflammatory adipokines [36]. Furthermore, leptin negatively modulates regulatory T cells and increases Th1 proliferation (leading to increased interferon-γ production); both of these effects are associated with airway hyperresponsiveness and airflow obstruction [37]. Leptin also stimulates the release of vascular endothelial growth factor (VEGF) from airway smooth muscle cells. VEGF may in turn promote subepithelial neovascularization and vascular permeability, which are important findings related to the pathogenesis of asthma [37].

Sex steroid hormones are likely involved in the sex-related serum leptin differences observed in the current study [38–40]. In murine adipocytes, leptin production and secretion were reduced by dihydrotestosterone but increased by 17β-estradiol. [39]. Increased leptin production per unit mass of adipose tissue induced by sex steroids may explain the higher serum leptin levels in women [38–40]. Conversely, sex steroids do not appear to influence resting gas exchange [41]. Thus, a greater amount of metabolically active adipose tissue in males may explain the differences in gas exchange previously reported between obese men and women [20,22]. The lower PaO_2_^POST^ observed in men in our study may likewise be explained by these differences in adipose tissue, as well as the lower serum leptin levels in men.

Obesity is one of the most important factors contributing to upper airway changes [42]. Fat deposition in the soft tissue of the pharynx increases extraluminal pressure and augments the mechanical load on the upper airway, thereby promoting upper airway narrowing and collapse. In addition, central adiposity decreases lung volumes by diaphragm displacement, further increasing pharyngeal collapsibility [43]. Postoperative upper airway or pharyngeal dysfunction and muscle weakness following anesthesia are additional factors that promote upper airway obstruction in obese patients, particularly those with OSA [1–3]. Animal models have demonstrated that leptin prevents upper airway obstruction through both peripheral mechanical and central neuromuscular actions [7]. Elevated serum leptin concentrations have been associated with increased neuromuscular responses of the upper airway during sleep in obese women scheduled for bariatric surgery [15]. Shapiro et al. hypothesized that leptin may enhance compensatory neural mechanisms triggered by upper airway obstruction, thereby reducing collapse of the upper airway and severity of OSAHS [15]. These responses were independent of BMI and may be insufficient if upper airway mechanical loads (passive pharyngeal critical pressure) are markedly elevated [13,15]. Using the *ob/ob* mouse model, Yao et al. identified activation of the forebrain (potentially the dorsomedial hypothalamus) as the mechanism through which leptin reduces upper airway obstruction during episode of sleep apnea [44].

Leptin may also play a role in positively modulating respiration. Models of animals lacking the gene responsible for leptin production exhibit marked abnormalities in breathing control, leading to respiratory failure (hypoxemia and hypercapnia) [7]. Although the precise mechanisms by which leptin exerts its respiratory effects remain under investigation [7], strong evidence suggests important roles of the central nervous system, including the brain’s melanocortin system [8]. Leptin increases the ventilatory response to CO_2,_ which is likely mediated by its action in hypothalamic and brainstem nuclei. In the hypothalamus, leptin's ventilatory effects appear to be mediated by the melanocortin system [8]. In the *ob/ob* mouse model, Yao et al. identified the hindbrain (possibly the nucleus tractus solitarius) as the main site of leptin’s effects on ventilatory control [44]. In humans, limited evidence has reinforced the relationship between leptin and ventilatory function in obesity. High leptin levels and central leptin resistance observed in OHS have been associated with hypoxemia and hypercapnia [14]. In hypercapnic obese patients, noninvasive ventilation significantly reduced serum leptin levels. These findings suggest that leptin may compensate for the increased ventilatory load in obesity by maintaining alveolar ventilation, and that noninvasive ventilation may reduce the need for high leptin levels to counteract the increased load [45].

This study has two main limitations. It is not a randomized controlled study and thereby has the drawbacks of all observational studies. Furthermore, several factors, including aspects of anesthetic care, may have influenced the postoperative data. Rapid, short-acting volatile anesthetics, low doses of opioids, sugammadex for reversing rocuronium-induced neuromuscular blockade, and prophylactic intraoperative lung-protective mechanical ventilation have been previously reported to improve postoperative respiratory function and gas exchange [46–48]. However, when using a standardized approach incorporating these strategies (as in this study), it appears that high leptin levels are associated with improved respiratory function of morbidly obese patients in the postoperative period.

## Conclusions

Although the precise mechanisms by which leptin affects respiratory function are not yet established, our data suggest that leptin is involved in respiration and may exert a stimulatory ventilatory effect in obese patients undergoing bariatric surgery. Leptin appears to have dual effects, as it is associated with worse preoperative oxygenation but improved respiratory function after surgery.

## Supporting information

S1 Data (PDF)
