# Supplementary material for: Dual effects of leptin in perioperative gas exchange of morbidly obese patients
Source: PLoS One. 2018 Jul 5;13(7):e0199610. doi: 10.1371/journal.pone.0199610 (PMC6033419; doi:10.1371/journal.pone.0199610)
Supplement: S1 Data — (PDF) [file pone.0199610.s001.pdf]

n	Age	Sex	BMI	LEPT	IL6	PaO2_pre
1	47	M	45	34	2,5	71,1
2	58	F	45,5	42	10,1	72,3
3	46	F	44,4	28	2,2	90
4	41	M	39,5	29	5,9	86,3
6	44	M	45	32	2,8	84,4
7	57	F	40	53	3	86,4
9	51	F	46,7	50	4,3	78,3
10	52	F	36,4	64	3,2	96,8
11	58	M	49,5	13	4,3	79,8
12	53	F	54,9	88	4	64,7
13	51	M	47,7	16	3	72
14	40	F	55,1	47	2,2	80
15	40	F	47,7	59	1,5	83,5
16	24	F	45,7	64	7,5	97
17	36	F	40,4	43	1,5	94,3
18	29	F	51,2	30	3,6	76,9
19	43	F	46,8	34	2,9	82,1
20	41	M	40,2	24	3,6	77,8
21	38	F	44,3	64	2,4	82
22	51	F	46,4	52	4	97
23	45	M	57,7	44	3,2	84,8
24	56	F	49,2	29	3,4	74,7
25	54	F	35,7	42	2,7	72,3
26	48	F	41	48,4	3,8	94,9
27	42	F	46,8	39	11,3	86,2
28	41	F	51,6	62	3,5	73,9
29	59	F	41	43	4,1	80,2
30	43	M	44,7	17	1,9	70,5
31	49	M	43,7	28	6	68,3
32	53	M	51,3	42	4,1	73
33	46	F	39,2	35	4,2	90
34	60	F	45,6	29	4,3	75,4
35	37	F	37,8	21	3,7	78
36	42	M	46,9	44	7,1	63,6
37	33	F	43,1	35	3,7	87,2
38	56	F	36,5	50	2,6	94,6
39	45	M	38,9	12	3,1	84,5
40	48	M	41,6	17	1,5	80,3
41	46	F	59,6	31	5,3	88,1
42	45	F	50,7	43	3,7	84,8
43	52	F	43,4	48	2,5	94,4
44	35	F	66,2	49	5,9	70,5
45	59	F	44,8	44	3,6	76,1
46	33	F	39,5	56	3,3	81,9

47	47	F	46,2	48	2,3	70,5
48	46	F	41,4	41	3,8	72,5
49	50	F	40,2	47	3,3	72,3
50	50	F	52,4	53	5	73,4
51	54	F	50,6	64	6,4	87,4
53	48	F	49,7	27	7	64
54	66	F	35,8	38	3,6	80
55	43	F	43,7	31	1,9	74,4
56	28	F	35,6	34	2,3	100,3
57	40	F	41	17	1,5	87,4
58	45	F	36,2	39	2,6	89,9
59	51	F	43,8	45	3,7	82,2
60	52	F	36,9	28	1,8	76,2
61	49	F	38,7	49	3,9	98,9
63	52	F	42,2	39	2,4	78,8
64	33	F	47,6	51	4,2	53,6
65	47	F	38,2	28	1,8	86,3
66	44	F	37	31	3,1	89,5
68	46	F	45	52	5,3	65,8
69	58	M	45	19	1,7	78
70	46	F	42	23	1,5	76,3
71	36	F	38	34	2,9	83,1
72	48	M	36	18	1,9	86,8
73	37	F	45	39	3,4	71,2
74	63	M	36,3	14	1,5	79,9
75	49	F	44	42	1,5	72,5
76	34	F	41	48	5,9	70,3
77	50	F	48,5	41	2,9	72,9
78	43	F	40	26	2,3	84,8
79	30	F	49,5	39	2,5	68,7
80	39	F	62	32	3,7	75,1
81	59	F	47,5	27	6,8	91,2
83	51	F	43	64	7,7	70,4
84	32	F	53	47	4,3	68,1
85	36	M	41	38	1,8	71,9
86	52	M	44,6	34	2,9	81,3
87	57	F	42,3	25	1,5	82,1
88	49	F	41,3	47	5,9	61,2
89	68	F	45,4	39	4,4	79,6
90	49	F	41	45	4,5	71,4
91	46	F	47	65	8,8	67,8
92	49	F	41,5	40	3,8	69,7
93	46	M	45,5	20	2,6	85,7
94	46	F	41,9	50	5,7	79,2
95	54	M	54,2	48	7,4	71,8

96	43	M	47	20	2,1	87,1
97	46	M	43,4	33	2,7	71,2
98	47	M	50,9	18	2,9	88,9
99	48	M	47,8	48	6,9	68,6
100	47	M	40	43	5,3	71,9
101	61	M	39,3	14	2,2	89,9
102	49	M	45,1	21	3,1	73,6
103	44	M	50,8	48	4,3	68,9
104	52	M	44,6	44	3,9	74,8
105	42	M	51,6	41	2,9	72,6
106	46	M	45,6	20	2,6	82,3
107	47	M	46,9	53	3,9	67,7
108	53	M	48,6	49	4	69,8
109	30	M	45,2	40	3,9	76,8
110	53	M	47,8	54	5,6	69,5
111	54	M	50,5	36	3,8	71,7
112	38	M	51,2	48	9,3	66,3
113	55	M	34,7	14	1,5	88,9
114	49	M	47,7	22	3,4	85,9
115	45	M	46,6	12	1,5	84,5
116	45	M	42,8	40	1,9	71
117	54	M	43,8	41	2,5	73,6
118	56	M	35,8	17	1,6	84,1

PaCO2_Pre	PaO2_post	PaCO2_Post	Delta_PaO2	Delta_PaCo2	LEPT_div
38,3	69	35,5	-2,1	-2,8	0
44,3	112,2	26,8	39,9	-17,5	1
32,6	84,8	43,7	-5,2	11,1	0
32,6	78	40	-8,3	7,4	0
35,1	74,5	45,5	-9,9	10,4	0
51,8	80,4	48,4	-6	-3,4	1
37,8	88,3	40,3	10	2,5	1
36	70,4	43	-26,4	7	1
36,9	81,9	36,9	2,1	0	0
39,7	72,3	48,8	7,6	9,1	1
46,1	55,7	47,1	-16,3	1	0
39	79,5	37,9	-0,5	-1,1	1
34,4	88,1	32	4,6	-2,4	1
34,1	93,4	41,9	-3,6	7,8	1
29,3	83,3	39,4	-11	10,1	1
34	85,9	40,3	9	6,3	0
33,6	86,2	37	4,1	3,4	0
32,8	92,8	33,5	15	0,7	0
30,8	108,8	36,5	26,8	5,7	1
34,1	93,4	41,9	-3,6	7,8	1
35,4	91,8	36,5	7	1,1	1
41	69,4	31,2	-5,3	-9,8	0
38	73,4	45,7	1,1	7,7	1
33,4	89,8	37,1	-5,1	3,7	1
37,8	73,2	42,2	-13	4,4	0
32,5	91,2	38,8	17,3	6,3	1
30,7	77,9	29,8	-2,3	-0,9	1
41,7	79	41,4	8,5	-0,3	0
41,1	63,6	44,9	-4,7	3,8	0
38,4	79,9	38,9	6,9	0,5	1
32,6	84,8	43,7	-5,2	11,1	0
33,8	102,9	29,8	25,5	-4	0
35,6	87	35,1	9	-0,5	0
38,8	61	48,7	-2,6	9,9	1
31,8	79,1	39,7	-8,1	7,9	0
36,7	82,9	46,4	-11,7	9,7	1
32,2	70,9	38,8	-13,6	6,6	0
37,1	83	41,8	2,7	4,7	0
35,6	82,2	42,7	-5,9	7,1	0
35,4	91,8	36,5	7	1,1	1
36,4	88,5	42	-5,9	5,6	1
41,7	79	41,4	8,5	-0,3	1
37,1	100,4	41,9	24,3	4,8	1
33,7	108,8	23,2	26,9	-10,5	1

41,7	79	41,4	8,5	-0,3	1
34,9	67,4	43	-5,1	8,1	1
44,3	112,2	26,8	39,9	-17,5	1
34	74	42,9	0,6	8,9	1
33,5	97,7	24,1	10,3	-9,4	1
35,5	61,4	43	-2,6	7,5	0
30,4	102,8	33	22,8	2,6	0
40,8	83,2	31,9	8,8	-8,9	0
24,7	88,8	37	-11,5	12,3	0
32,5	69,1	38,9	-18,3	6,4	0
31,5	91,4	44,6	1,5	13,1	0
42,8	75,6	40,9	-6,6	-1,9	1
36	69,5	49,7	-6,7	13,7	0
29	88,9	35	-10	6	1
40	69,2	49,2	-9,6	9,2	0
40,1	82,3	44,1	28,7	4	1
36,4	82,2	25,7	-4,1	-10,7	0
34,2	88,9	41,4	0,6	7,2	0
35,6	64,4	40,8	-1,4	5,2	1
45,6	77,2	43,8	-0,8	-1,8	0
42,1	79,1	43,2	2,8	1,1	0
33,4	76,5	39,9	-6,6	6,5	0
38	72,8	39,4	-14	1,4	0
38,9	79,9	36,7	8,7	-2,2	0
38,7	78,7	38,7	-1,2	0	0
36,9	77,2	39,2	4,7	2,3	1
38,8	74,4	40,6	4,1	1,8	1
40,6	86,5	37,6	13,6	-3	1
41,1	83	42,6	-1,8	1,5	0
42,4	66,1	43,2	-2,6	0,8	0
38,9	91,2	40,2	16,1	1,3	0
40,2	92,7	39,9	1,5	-0,3	0
41,5	86	43,1	15,6	1,6	1
42,3	81,9	46,2	13,8	3,9	1
43,2	67,1	39,6	-4,8	-3,6	0
38,8	75,1	39,7	-6,2	0,9	0
41,2	73	42,4	-9,1	1,2	0
40,3	66,7	34,8	5,5	-5,5	1
47	51,4	50,1	-28,2	3,1	0
41,1	83,5	39,3	12,1	-1,8	1
37,9	77,9	35,8	10,1	-2,1	1
40,1	71,1	39,8	1,4	-0,3	1
42,4	83	47,6	-2,7	5,2	0
39,1	85,1	36,1	5,9	-3	1
40,2	79,4	38,7	7,6	-1,5	1

36,5	89,8	38,2	2,7	1,7	0
36,3	72,6	39,4	1,4	3,1	0
39,6	92,9	41,8	4	2,2	0
38,7	79,7	36,5	11,1	-2,2	1
38,6	83,5	37,2	11,6	-1,4	1
39,7	87,1	41,1	-2,8	1,4	0
41,2	68,9	42,9	-4,7	1,7	0
38,8	78,3	35,7	9,4	-3,1	1
37,4	81,8	36,5	7	-0,9	1
42,3	77,9	35,9	5,3	-6,4	1
41,3	77,5	43,4	-4,8	2,1	0
42,1	87,1	37,9	19,4	-4,2	1
40,6	72,4	38,8	2,6	-1,8	1
40	69,2	44,2	-7,6	4,2	1
40,8	66,6	36,5	-2,9	-4,3	1
38,1	65,6	41,7	-6,1	3,6	0
41,3	72,3	44,2	6	2,9	1
35,6	92,4	43,4	3,5	7,8	0
36,2	78,9	41,3	-7	5,1	0
36,7	74,4	41,8	-10,1	5,1	0
43,6	78,2	42,8	7,2	-0,8	1
38,1	71,9	42,3	-1,7	4,2	1
34,3	78,5	38,9	-5,6	4,6	0

Delta_PaO2 DIV	Delta_PaCo2 DIV
0	0
1	0
0	1
0	1
0	1
0	0
1	1
0	1
1	1
1	1
0	1
0	0
1	0
0	1
0	1
1	1
1	1
1	1
1	1
0	1
1	1
0	0
1	1
0	1
0	1
1	1
0	0
1	0
0	1
1	1
0	1
1	0
1	0
0	1
0	1
0	1
0	1
0	1
0	1
1	1
0	1
1	0
1	1
1	0

1	0
0	1
1	0
1	1
1	0
0	1
1	1
1	0
0	1
0	1
1	1
0	0
0	1
0	1
0	1
0	1
0	0
0	1
0	1
0	0
1	1
0	1
0	1
1	0
0	1
1	1
1	1
1	0
0	1
0	1
1	1
1	0
1	1
1	1
0	0
0	1
0	1
1	0
0	1
1	0
1	0
1	0
0	1
1	0
1	0

1	1
1	1
1	1
1	0
1	0
0	1
0	1
1	0
1	0
1	0
0	1
1	0
1	0
0	1
0	0
0	1
1	1
1	1
0	1
0	1
1	0
0	1
0	1
